# Prevalence and Clinicopathologic Features of Canine Metastatic Melanoma Involving the Central Nervous System: A Retrospective Analysis and Comparative Review

**DOI:** 10.3389/fonc.2022.868004

**Published:** 2022-05-27

**Authors:** Aryana M. Razmara, Luke A. Wittenburg, Sami Al-Nadaf, Ryan G. Toedebusch, Frederick J. Meyers, Christine M. Toedebusch

**Affiliations:** ^1^ Department of Surgical and Radiological Sciences, School of Veterinary Medicine, University of California, Davis, Davis, CA, United States; ^2^ UCD Comprehensive Cancer Center, Sacramento, CA, United States; ^3^ Department of Internal Medicine, Division of Hematology and Oncology, Center for Precision Medicine, University of California, Davis School of Medicine, Sacramento, CA, United States

**Keywords:** malignant melanoma, central nervous system, canine, brain, seizure, large-animal model

## Abstract

**Background:**

Central nervous system (CNS) involvement is the leading cause of death in malignant melanoma. Rodent models, while vital to mechanistic investigation, have had limited success identifying effective therapies for melanoma brain metastases. The companion dog with *de novo* melanoma is a promising complementary model for developmental therapeutic investigation, as these tumors occur in an immunologically outbred host that has shared environmental exposures with humans. However, relatively little is known regarding the prevalence and clinicopathological features of canine melanoma metastasis to the CNS. To further validate the dog as an appropriate model for human metastatic melanoma, the aims of this study were to determine the rate of CNS metastasis and associated clinicopathologic features in canine malignant melanoma.

**Methods:**

Medical records of dogs diagnosed with malignant melanoma from 1985-2019 at the University of California Davis Veterinary Medical Teaching Hospital were assessed retrospectively. Clinicopathologic features were compared between dogs with CNS metastasis (CNS+) and dogs without CNS metastasis (CNS-). Site of CNS involvement and associated neurological signs were analyzed *via* Wilcoxon-Mann-Whitney rank sum and Fisher’s exact tests. Survival data were analyzed *via* Kaplan-Meier estimates.

**Results:**

CNS metastasis was identified in 38% of dogs in this study (20/53). The oral cavity was the most common site of primary melanoma in both groups [CNS+: n=12 (60%) vs. CNS-: n=22 (67%); p>0.99]. The total burden of metastatic disease was higher in the CNS+ group (CNS+: 4, 95% CI 3-5 vs. CNS-: 3, 95% CI 1-3; p<0.001). The cerebrum was the most common site of CNS metastasis (n=15, 75%) and seizures were the most observed neurological sign (n=9, 64%). There was no difference in overall survival between CNS+ and CNS- groups. However, the median survival time following onset of neurological signs was 9.5 days (95% CI 1-43), with 5 dogs euthanized within 24 hours of the onset of neurological signs.

**Conclusions:**

Canine and human MM patients share similar rates of CNS metastasis and clinical presentation. This study will guide clinical management of canines with malignant melanoma and inform future studies using dogs with spontaneously occurring melanoma as a preclinical model for human melanoma brain metastases.

## Introduction

Nearly 100,000 new cases of melanoma are diagnosed in the United States each year (1, 2) with incidence rates increasing by 1.8% each year in adults over 40 (3). While the rate of metastasis is low (2-8%), those melanomas that are thicker, deeper, and invade into the vasculature pose a greater risk for metastases. In particular, malignant melanoma has a high risk for brain metastasis (4, 5), with the brain as a site of dissemination in as many as 80% of patients with metastatic disease (6). Uniformly fatal, the median survival for patients with melanoma brain metastasis (MBM) is approximately four months following diagnosis. Central nervous system (CNS) involvement is the leading cause of death from this disease (7, 8), demonstrating an urgent need for an increased understanding of molecular and cellular mechanisms contributing to brain invasion.

While recent immunomodulatory and targeted therapeutic advances have increased MBM response rate, overall survival (OS) remains considerably shorter compared to patients with extracranial metastases (9–11). There are myriad challenges that constrain successful treatment of MBM. The blood-brain barrier, which precludes effective delivery of many drugs (12, 13), also alters metastatic seeding (14, 15). In fact, there is growing evidence that MBM has a unique molecular signature relative to primary and extra-cranial melanoma metastases (16, 17), including a higher mutational burden and increased PD-L1 expression. Unfortunately, current preclinical models that utilize direct intracranial implantation or *in vitro* selection of brain-seeking clones, do not recapitulate this regional tumoral heterogeneity, nor the molecular cascade required for brain colonization. Thus, rodent models, while vital to mechanistic investigation, have had limited success in identifying effective therapies for MBM (18). The companion dog with naturally occurring metastatic melanoma is a promising complementary model for comparative studies and developmental therapeutic investigation, as these tumors arise *de novo* in a host that is immunologically outbred and has shared environmental exposures with humans (19, 20).

Naturally occurring canine melanoma accounts for 7% of all malignant cancers in dogs (21, 22) and recapitulates striking similarity to human mucosal melanoma (23). In contrast to cutaneous melanoma, which occurs infrequently and metastasizes in less than 25% of cases, (24), dogs most commonly develop oral melanoma (23, 25). Like human mucosal melanoma, tumors most commonly occur on the gingiva, oropharynx, and palate and are highly locally invasive. Moreover, thick, deep tumors with vascular invasion are risk factors for metastases, which occurs in 70-90% of canine cases (23). Importantly, canine and human melanoma share a similar genetic landscape, as demonstrated by constitutive ERK activation (26) and somatic mutations in NRAS and PTEN (27).

Brain metastasis is a feature of canine melanoma (28), thus providing a unique platform to investigate novel therapeutics targeting CNS metastasis. However, relatively little is known regarding the prevalence and clinicopathological features of canine melanoma CNS metastasis, exposing a critical knowledge gap for translational applications. A high neutrophil to lymphocyte ratio (NLR) in the peripheral blood has been associated with worse overall survival and progression-free survival in human patients with metastatic melanoma (29), and has been shown to be predictive of brain metastases in non-small cell lung cancer (30). The relationship of these clinicopathologic parameters to CNS involvement in canine melanoma has not been evaluated. Therefore, this study aimed to examine the rate of CNS metastases and clinicopathologic features of MBM in dogs and compare these features to the human disease.

## Methods

### Study Population

This was a retrospective, investigational study conducted at the University of California (UC) Davis School of Veterinary Medicine, Veterinary Medical Teaching Hospital (VMTH). Medical records from 1985 through 2019 were reviewed for cases with a primary inclusion criteria of a histopathological diagnosis of malignant melanoma (31). At our institution, it is standard practice to perform a gross examination of sectioned brain in all cases of systemic neoplasia, particularly those with a high tendency for metastasis. Thus, all cases included in this study had a gross examination of sectioned brain +/- the spinal cord, followed by histology if a lesion was noted. Dogs without gross examination of sectioned brain were excluded from the study population. Cases were divided according to the presence or absence of metastasis to the CNS. The CNS positive group (CNS+) included cases with metastasis to the brain and/or spinal cord. The CNS negative group (CNS-) included cases with either no evidence of metastasis or confirmation of extra-CNS metastasis. Cases were excluded if the dog was alive at the time of data collection, or the dog did not have clinical signs related to melanoma prior to death or humane euthanasia.

### Clinicopathological Parameters

Clinical data from all cases were extracted from the medical record. Dog breed, sex, age at time of diagnosis, site, and size of primary tumor (if known), as well as location of metastases were recorded. Primary tumor locations were categorized as follows: oral (tongue, buccal mucosa, palate), digital, cutaneous, or ocular. Of the dogs with CNS involvement, the number and location of CNS metastases, neurological signs, and complete neurological examination findings were recorded.

Complete blood counts (CBC) and serum chemistry data were included from cases in which samples were acquired at UC Davis VMTH within three weeks of the initial diagnosis and prior to specific treatment. Neutrophil, lymphocyte, and platelet counts were recorded and NLRs were calculated. Additionally, serum calcium and albumin concentrations were recorded. Data from cases that received corticosteroids within one week prior to blood collection were excluded.

### Treatment and Outcome

Definitive treatment was defined as specific treatment modalities including surgical resection, radiation treatment, chemotherapy, or immune therapy, used alone or in combination. Palliative treatment was defined as treatment with non-steroidal anti-inflammatory, corticosteroid, and/or analgesic medication.

Survival was defined as the time from diagnosis of malignant melanoma to humane euthanasia. Of the dogs with CNS metastasis, time from diagnosis of malignant melanoma to onset of neurological signs was also recorded. However, if there was an unknown date of diagnosis (n=2), or the onset of neurological signs occurred prior to date of diagnosis (n=2), cases were excluded from survival analysis.

### Statistical Analysis

Data sets were tested for normal distribution using the D’Agostino-Pearson normality test and inspection of normal probability plots. Continuous variables were compared using the Wilcoxon rank sum (non-normal distribution) or paired T-test (normal distribution) to determine whether clinical data and outcome measures varied between dogs with and without CNS metastasis. Frequency of categorical variables were compared between dogs with and without CNS metastasis using Fisher exact testing. Association between variables was assessed using the nonparametric Spearman correlation coefficient. Kaplan-Meier estimates of median survival were compared using Mantel-Cox Log-rank analysis. When applicable, tests were two-tailed with a 95% confidence interval and P values < 0.05 were considered significant. All statistical analyses were performed with GraphPad Prism version 8.4.3 (GraphPad Software, San Diego, California USA).

## Results

### Patient Characteristics

**Figure 1:**
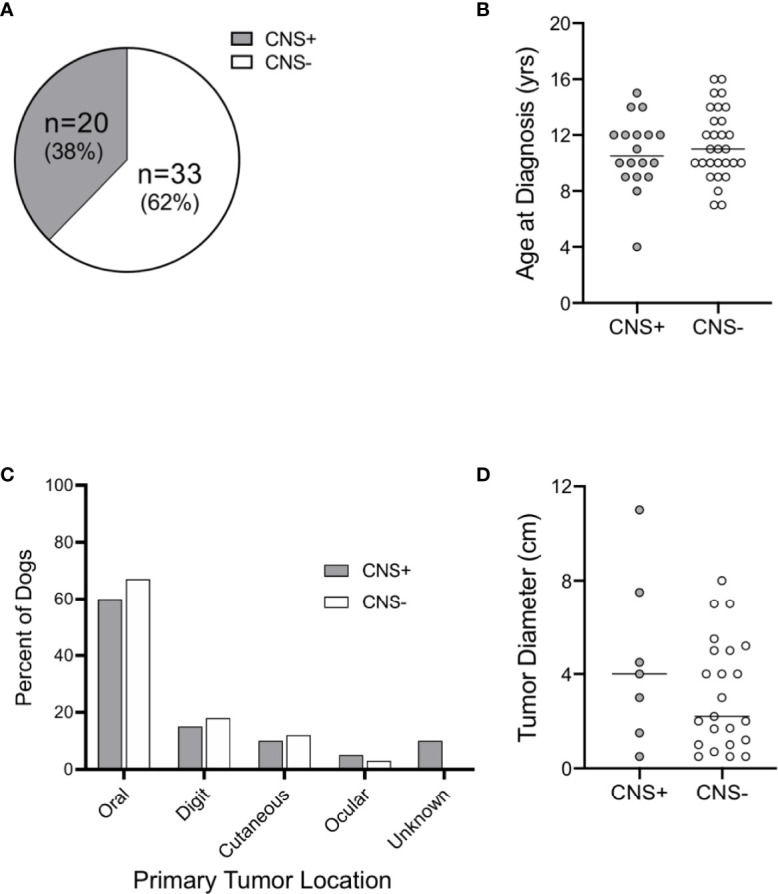
Distribution, age, and primary tumor characteristics of dogs with malignant melanoma. **(A)** Distribution of dogs with central nervous system (CNS) metastasis of malignant melanoma at the time of necropsy. **(B)** Median age at diagnosis was similar between groups. Primary tumor **(C)** distribution and **(D)** diameter were similar between CNS+ and CNS- dogs (primary tumor site was not determined for 2 CNS+ dogs). Comparisons based on Wilcoxon-Mann-Whitney rank sum test.

Fifty-three dogs were identified with a histopathological diagnosis of malignant melanoma during the study period and met the criteria to be included in the study cohort. Approximately 38% of dogs had CNS metastases (CNS+; n=20) vs. 62% of dogs without CNS metastases (CNS-; n=33); **Figure 1A**). The study included 41 purebred and 12 mixed breed dogs (**Supplementary Table 1**). Relative to the general hospital population, the Rottweiler (0.9% vs. 10%, n=2) and Chow Chow (0.1% vs. 10%, n=2) were over-represented in the CNS+ group. Labrador retrievers were over-represented in the CNS- group (2.6% vs. 15.2%, n=9), followed by the Rottweiler (9.1%, n=3), Boxer (0.4% vs. 9.1%, n=3), Chow Chow (6.1%, n=2), Shih Tzu (0.3% vs. 6.1%, n=2), and Golden Retriever (1.2% vs. 6.1%, n=2). Age at diagnosis was similar between groups (CNS+: 10.5 years, CI 9-12 vs CNS-: 11 years, CI 10-13; p = 0.521) (**Figure 1B**).

### Primary Tumor Characteristics

Two dogs in the CNS+ group had unconfirmed primary tumor sites. Of the dogs with known primary tumor sites, the oral cavity was the most common site of primary melanoma in both groups [CNS+: n=12 (60%) vs. CNS-: n=22 (67%); p>0.99] (**Figure 1C**). Digital, cutaneous, and ocular tumors comprised the remaining sites with similar frequency between groups (p=0.98). Instead of tumor thickness and depth of invasion, primary tumor diameter was recorded; tumor diameter was not different between groups (CNS+: 4.0 cm, 95% CI 0.5-11 vs. CNS-: 2.2 cm., 95% CI 1.2-5; p=0.45) (**Figure 1D**).

### Clinical Variables Predictive of CNS Metastases

**Figure 2:**
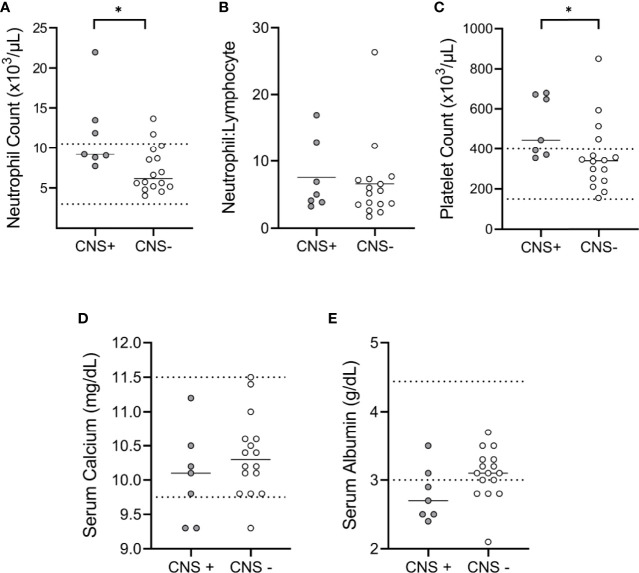
Clinicopathologic features of canine malignant melanoma. CBC and chemistry panel data were available for comparison between CNS+ (n=7) and CNS- (n=16) dogs. **(A)** The median neutrophil count was higher in CNS+ dogs (p= 0.015). **(B)** Neutrophil to lymphocyte ratios were not different between groups (p= 0.579). **(C)** The median platelet count for CNS+ dogs was higher than CNS- dogs (p= 0.018). No differences were observed in **(D)** serum calcium (p=0.299) or **(E)** albumin (p=0.077) between groups. Dashed lines represent hospital normal reference ranges. Comparisons based on Wilcoxon-Mann-Whitney rank sum test. *p<0.05.

CBC and serum chemistry analyses were available for 7 CNS+ (35%) and 16 CNS- (48%) cases. The median neutrophil count (9173, 95% CI 7712-21943 vs. 6151, 95% CI 5161-9811; p=0.02) (**Figure 2A**) and the median platelet count (444, 95% CI 353-680 vs. 341, 95% CI 241-448 x 10^3^/µL; p<0.02) (**Figure 2C** **)**, were higher in CNS+ dogs. However, the neutrophil:lymphocyte ratio (**Figure 2B**; p=0.58), serum calcium (**Figure 2D**; p=0.30), and serum albumin (**Figure 2E**; p=0.08) were similar between groups.

**Figure 3:**
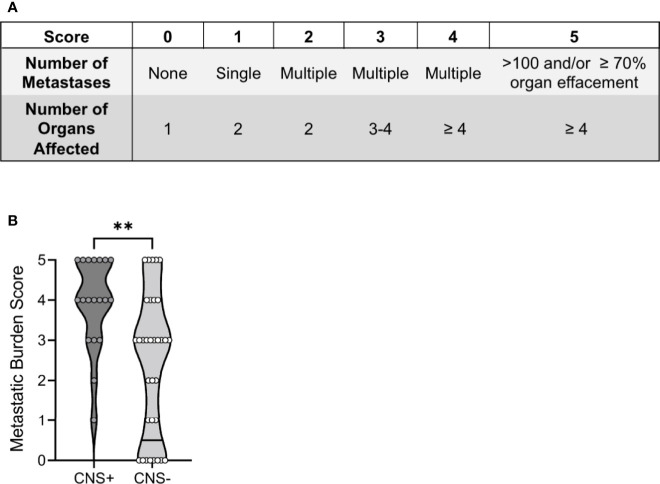
Total burden of CNS and extra-CNS metastases in dogs with malignant melanoma. **(A)** The metastatic burden scoring system comprised the number of 1) metastases and 2) organs affected. **(B)** The median metastatic burden score was higher in CNS+ (n=19) dogs (p=0.0014). One CNS+ dog did not have an extra-neural post-mortem examination and therefore could not be assigned a score. Comparison based on Wilcoxon-Mann-Whitney rank sum test. **p<0.01.

To compare 1) the number of metastases and 2) the number of organs affected between groups, we developed a metastatic burden score (0-5; **Figure 3A**), with 0 representing a single organ affected/no metastasis and 5 representing >100 metastases or 70% organ effacement/≥4 organs affected. This scoring classification was created based on the tumor burden score (TBS), which uses tumor size and number to successfully predict prognosis in human cancers (32–34), as well as the metastatic site index (MSI), which incorporates the number of metastatic systems (35). Notably, 8/33 (24%) CNS- dogs scored 0, while fifteen (75%) of CNS+ dogs had a score ≥4. Correspondingly, the metastatic burden score was higher in CNS+ dogs (4, 95% CI 3-5 vs. CNS-: 3, 95% CI 1-3; p<0.001) (**Figure 3B**). The most common site of extra-CNS metastasis was the lung, followed by lymph nodes. The heart, kidney, and adrenal glands were also affected, most commonly in the CNS+ group (**Supplementary Table 2**).

### Neurological Signs and CNS Site(s) of Metastasis

**Figure 4:**
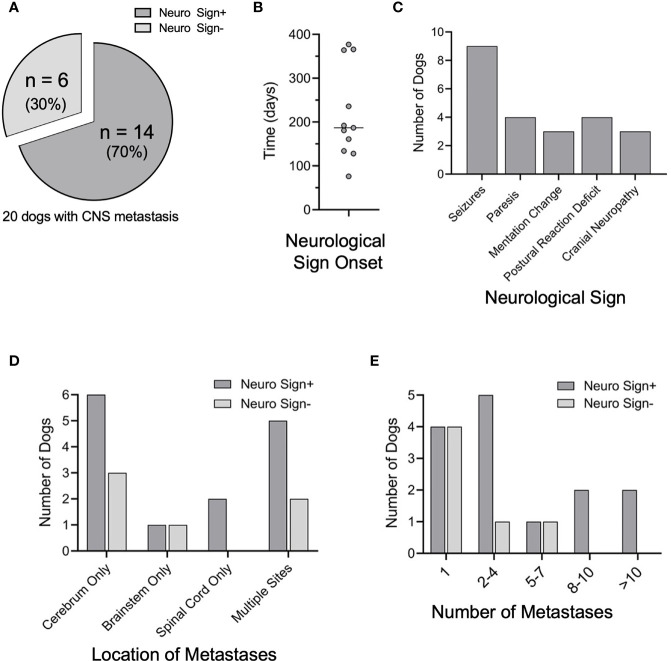
Neurological signs and CNS disease burden. **(A)** Of the 20 CNS+ dogs, 14 developed neurological signs prior to humane euthanasia or death. **(B)** Elapsed time between the initial diagnosis of melanoma and the onset of neurologic signs (n=11). **(C)** Of those that developed neurological signs, the most common initial sign noted was seizures, followed by paresis, postural reaction deficits, mentation change, and cranial nerve neuropathy. **(D)** Although location of CNS metastases varied among CNS+ dogs, the cerebrum was the most common site of metastases. Location of metastases were similar between dogs with and without neurological signs (cerebrum p=0.642; brainstem p=0.521, spinal cord p=0.999). **(E)** The number of CNS metastases between dogs with and without neurological signs was not different (1, p=0.161; 2-4, p=0.613; 5-7, p=0.521; 8-10 and > 10, p= 0.999). Comparisons were made by two-tailed Fisher’s Exact tests.

Of the 20 dogs with CNS metastasis, 14 (70%) exhibited neurological signs (**Figure 4A**) within a median time of 171 days following primary tumor diagnosis (**Figure 4B**). The CNS disease burden was clinically silent and only identified at the time of necropsy in six dogs (30%). Seizures were the most common neurological sign observed (n=9, 64%) (**Figure 4C**), corresponding with the cerebrum as the most common site of metastasis in this group (n=10, 71%) (**Figure 4D** and **Supplemental Table 3**). Long tract signs (paresis, postural reaction deficits) were noted in 4 dogs (29%) (**Figure 4C**), with mentation changes and cranial nerve deficits observed in 3 dogs (21%) (**Figure 4C**).

Of all CNS+ dogs, the cerebrum remained the most common site of metastasis (n=15, 75%) (**Figure 4D** and **Supplemental Table 3**). The frontal lobe was the most common site of cerebral metastasis (n=5, 25%), followed by the temporal lobe (n=4, 15%) and occipital lobe (n=3, 15%). Notably, most dogs with clinically silent CNS involvement had metastases to the cerebrum (n=5, 83%). The brainstem was involved in 5 dogs (20%), with concurrent cerebral involvement in 3/5 of these dogs. The spinal cord was affected in 4 dogs, with 3/4 dogs exhibiting neurological deficits from their metastases. The three dogs with neurological signs had metastases to the thoracolumbar spinal segments or the 8^th^ cervical spinal segment and spinal nerve within the spinal canal. The fourth dog, without neurological signs, had metastasis to the cauda equina. The majority of the 14 dogs exhibiting neurological signs had multiple CNS metastases (n=10, 71%) (**Figure 4E**), with 4 dogs (29%) exhibiting ≥ 8 sites of metastases. Metastases were confined to the cerebrum in 7/14 dogs with multiple CNS metastases and neurological signs (**Supplementary Table 3** **).**

### Impact of CNS Metastasis on Treatment and Survival

**Figure 5:**
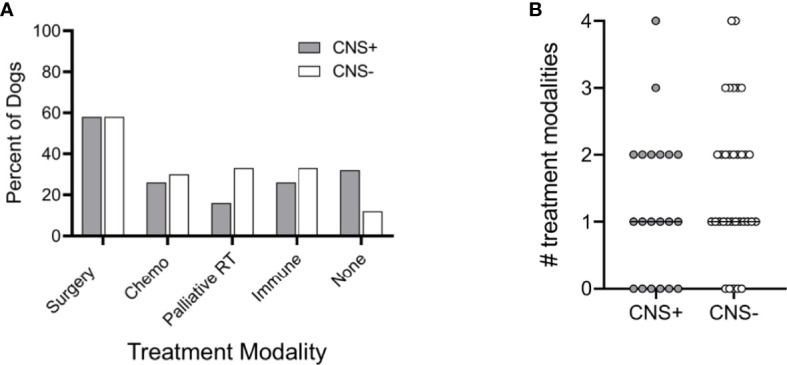
Treatment modalities utilized in dogs with melanoma. **(A)** Percentage of dogs treated with each modality, either alone or in combination, was similar between dogs that developed CNS metastasis and those that did not. **(B)** The median number of treatment modalities was not significantly different between groups (p=0.263). Comparison based on Wilcoxon-Mann-Whitney rank sum test. Chemo, cytotoxic chemotherapy; Palliative RT, palliative radiation therapy; Immune, immunotherapy (i.e., melanoma vaccine).

Dogs in each group had varying types of treatment for their primary tumor including surgery, chemotherapy, palliative radiation therapy, immune therapy (melanoma vaccine), or no treatment (**Figure 5A**). Surgery was the most common treatment in each group and ranged from debulking procedures to partial maxillectomies, mandibulectomies or digit amputation. Most dogs had some combination of treatment modalities. There was no difference in the type of treatment or number of treatment modalities between CNS+ and CNS- groups (**Figure 5B**; p=0.263).

**Figure 6:**
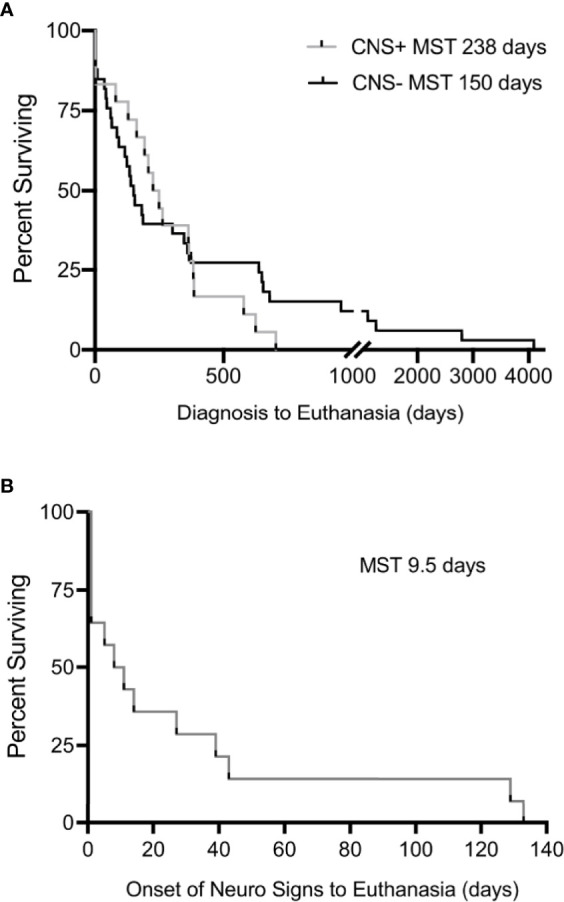
Kaplan-Meier estimates for **(A)** interval between initial diagnosis of malignant melanoma to euthanasia in CNS+ and CNS- dogs, demonstrating no significant difference in median survival (p=0.580). Dogs with unknown dates of diagnosis and dogs who exhibited signs prior to diagnosis were excluded. **(B)** The interval between onset of neurological signs to humane euthanasia was 9.5 days (n=14). Comparisons in survival made by Mantel-Cox Log-rank analysis.

Survival time following diagnosis was available for 49/53 cases (18 CNS+, 31 CNS-). Two dogs with unknown dates of melanoma diagnosis and two dogs that exhibited CNS signs prior to their diagnosis were excluded from survival analysis. Overall survival between CNS+ and CNS- dogs was not different (238 days, 95% CI 129-383 vs. 150 days, 95% CI 86-359; p=0.58) (**Figure 6A**). However, it should be noted that CNS+ dogs died or were euthanized within two years of diagnosis of melanoma. In contrast, five CNS- dogs (15%) survived longer than two years following diagnosis, three with cutaneous melanoma and two with digital melanoma. To evaluate the specific impact of neurological deficits on survival, dogs with neurological signs were evaluated separately. The median survival time following onset of neurological signs was 9.5 days (95% CI 1-43) (**Figure 6B**). Of these dogs, 5 (31%) were euthanized within 24 hours of the onset of neurological signs.

## Discussion

Our study defines the prevalence of CNS metastases in naturally occurring canine malignant melanoma, as well as the common sites of metastases and associated neurological signs. Based on this study population, canine and human malignant melanoma share similar rates and locations of CNS metastases. Thus, companion dogs with naturally occurring malignant melanoma may provide a complementary model for investigating novel therapeutic interventions for CNS metastatic melanoma.

Human malignant melanoma has a high tendency for CNS metastasis, with 8-46% of patients receiving a clinical diagnosis of CNS metastasis and upwards of 75% of patients diagnosed at the time of autopsy (6, 7). In this study population, we observed a similar prevalence of CNS metastases in dogs (38%). However, we noted clear differences in the primary tumor site between species. Cutaneous melanoma represents over 91% of human malignant melanoma cases (36). Primary tumors typically arise from the trunk/abdomen (7, 37, 38), although patients with CNS metastasis have been reported to have a higher prevalence of head and neck primary tumors (5). We and others (39) have demonstrated that approximately 60% of canine malignant melanoma arises from multiple sites within the oral cavity, including the tongue, gingiva, and palette. In contrast, primary oral malignant melanoma is rare in humans (40, 41) and typically originates on the maxilla (42).

Multiple risk factors for CNS metastases in human melanoma have been described. Primary tumor location of the head and neck is a key predictor of CNS metastases (30-40%) (5, 37). However, primary tumor location does not appear to be predictive of CNS involvement in canine melanoma, as we observed a similar distribution in primary tumor site between CNS+ and CNS- groups. Systemic inflammation has also been implicated in melanoma progression and metastasis. In particular, neutrophils are actively involved in cancer metastasis, whereby they facilitate cancer cell escape from the primary tumor and promote entry into the vasculature and extravasation into distant organs (43). Specifically, the NLR has been identified as a prognostic biomarker for many cancers (30, 44–46). In fact, an NLR ≥ 5, while rare in melanoma patients with a single metastatic site, was increasingly common in patients with multiple metastases (47). The relationship between NLR and CNS metastases is less clear, but a NLR >4.95 has been associated with brain metastases in non-small-cell lung cancer. In our study cohort, CNS+ dogs had increased peripheral blood neutrophil counts relative to CNS- dogs. Strikingly, we observed a median NLR ≥ 5 in both groups, with no observed difference between groups. This is consistent with previous data that demonstrated an elevated NLR in canine patients with metastatic histiocytic sarcoma, independent of CNS involvement (48). Thus, the NLR in canine cancer may represent a broad predictor of disease burden, rather than a specific biomarker for CNS dissemination.

Brain metastasis is considered the final stage of tumor progression in human malignant melanoma (49, 50), with a median time from initial diagnosis of melanoma to detection of intracranial metastasis ranging from 18 to 32 months (5, 37). However, canine melanoma may invade the CNS more quickly. In our study, the median time from melanoma diagnosis to a clinical diagnosis of CNS metastases was 171 days, with thirty percent of CNS+ dogs asymptomatic from their CNS disease. The clinical diagnoses in our study population were limited to the onset of neurological signs, with few cases receiving advanced brain imaging at the time of diagnosis. Unlike human medicine, there are no clear recommendations for advanced imaging of the CNS in canine melanoma (51). The National Comprehensive Cancer Network recommends that all Stage IV patients receive a contrast brain MRI or CT (52). Even with this recommendation, 15-40% of human patients are symptomatic from CNS disease at the time of diagnosis (38), suggesting that brain imaging should be considered earlier in these patients. Similarly, given the high prevalence of CNS metastases in canine melanoma, advanced brain imaging should be considered in all dogs with Stage III or IV melanoma to optimize early detection of CNS involvement for improved therapeutic strategies.

Of all brain metastatic neoplasia, melanoma carries the most risk for tumor-associated epilepsy (53). The presence of brain metastases in melanoma patients has been identified as a significant predictor of epileptic seizures (54). While not all symptomatic patients are affected by seizures, reported prevalence ranges from 0-67% (38, 55–57). Our data suggest that canine melanoma is also a risk factor for metastasis-related epilepsy. Seizures were the most common neurological sign in dogs with CNS metastases. In line with this observation, the cerebrum was the most common site of metastases, with the frontal lobe affected in 25% of all CNS+ dogs. Frontal lobe involvement, as well as the number of neurological symptoms, are negative prognostic indicators of survival in human metastatic melanoma (5). Moreover, approximately 2/3 of human patients with brain metastases will have two or more lesions (5, 37). Our data suggest a similar metastatic disease burden in canine melanoma, as two or more lesions were diagnosed in 55% of dogs with CNS metastases. In fact, 20% of dogs in this population had eight or more CNS metastatic lesions at the time of necropsy. The propensity of melanoma metastasis to the frontal lobe and clinical manifestation of seizures highlights a meaningful similarity between human and canine malignant melanoma and facilitates the translation of therapeutic interventions.

Across studies, the development of brain metastases has been associated with a poor prognosis and reduced overall survival in melanoma patients (5, 7, 37). We observed a striking effect of the presence of neurological signs on patient survival. The median survival time following onset of neurological signs to death was less than ten days, with 5/14 (36%) of dogs humanely euthanized within 24 hours. This is consistent with the reported survival time of a few weeks for human patients with untreated CNS disease (58, 59). Remarkably, this relationship was not reflected in overall patient survival following melanoma diagnosis, as we did not observe a difference in overall survival between dogs with and without CNS metastases in our study population. While our institution routinely examines all brains of dogs with malignant melanoma at necropsy, the spinal cord is only examined in cases with myelopathic signs. Therefore, it is possible that this lack of observed survival time between groups is in fact, Type II Error. Alternatively, this apparent contradiction may be the result of comparable cumulative disease burdens between CNS+ and CNS- dogs, which may indicate that cumulative disease burden, rather than CNS metastases, is more clinically determinant of survival in this species. Similarly, stage IV human melanoma, independent of location of metastasis, has been associated with a marked reduction in 5-year survival. However, when survival was stratified according to the site of initial metastasis, patients with brain metastases had the shortest survival time, with median survival of fewer than six months (60). This important difference likely highlights cultural rather than biological determinants of survival, as survival in dogs is generally determined by the owner’s decision for humane euthanasia.

Given the magnitude of the morbidity and mortality in human and canine melanoma patients, it is prudent to further define the mechanisms which facilitate CNS metastases. Recent evidence challenges the paradigm of brain metastases occurring as a late event following local disease progression. Circulating tumor cells have been detected in early-stage cancer (61), and it has been suggested that brain micrometastases form early in the disease course. The reciprocal interaction of melanoma cells and cells of the brain microenvironment is hypothesized to be a major determinant in brain metastasis formation (62). In particular, resident brain microglia have been shown to play a major role in supporting CNS metastases. Microglia surround and infiltrate human brain metastases (63), and rodent models have demonstrated a symbiosis between melanoma cells and microglia. Similar to what has been observed in glioblastoma (64–66), melanoma cells induce microglial proliferation and reprogram the microglial secretome to promote their own metastasis. Under the melanoma cell influence, microglia release soluble factors which increase melanoma proliferation and invasion into the brain parenchyma. Preliminary data from our laboratory indicate that microglia surround and infiltrate canine melanoma brain metastases (Consales et al., unpublished data), consistent with the microglial response in human patients. Thus, improved functional and molecular characterization of microglia in canine melanoma brain metastases may facilitate the development of novel therapeutic targets for canine and human patients alike.

## Conclusions

This retrospective study observed a high prevalence of CNS distribution in canine metastatic melanoma, similar to human metastatic melanoma. Moreover, canine metastatic CNS melanoma has similar clinicopathologic features to human disease, including a high NLR, seizures, and tendency for multiple sites of CNS metastases. While dogs may have a shorter disease interval, we have identified many key clinical similarities between species, further substantiating the parity of human and canine CNS metastatic melanoma. Therefore, the companion dog with naturally occurring metastatic melanoma may provide a valuable, complementary model to evaluate novel therapies for CNS metastases.

## Data Availability Statement

The raw data supporting the conclusions of this article will be made available by the authors, without undue reservation.

## Author Contributions

Conceptualization, AR, LW, and CT. Methodology, AR, LW, SA-N, RT, and CT. Investigation, AR. Writing—original draft preparation, AR and CT. Writing—review and editing, AR, LW, SA-N, RT, FM, and CT. Funding acquisition, LW and CT. All authors have read and agreed to the published version of the manuscript.

## Funding

This research was supported in part by the UC Davis Paul Calabresi Career Development Award for Clinical Oncology as funded by the National Cancer Institute/National Institutes of Health through grant #2K12CA138464-11 (CT). Additional support was provided by the NIH K01 OD026526 (LW) and the Paul C. and Borghild T. Petersen Brain Tumor Foundation.

## Conflict of Interest

The authors declare that the research was conducted in the absence of any commercial or financial relationships that could be construed as a potential conflict of interest.

## Publisher’s Note

All claims expressed in this article are solely those of the authors and do not necessarily represent those of their affiliated organizations, or those of the publisher, the editors and the reviewers. Any product that may be evaluated in this article, or claim that may be made by its manufacturer, is not guaranteed or endorsed by the publisher.
