# Supplementary figures and images for: Prevalence and Clinicopathologic Features of Canine Metastatic Melanoma Involving the Central Nervous System: A Retrospective Analysis and Comparative Review
Source: Front Oncol. 2022 May 27;12:868004. doi: 10.3389/fonc.2022.868004 (PMC9186031; doi:10.3389/fonc.2022.868004)

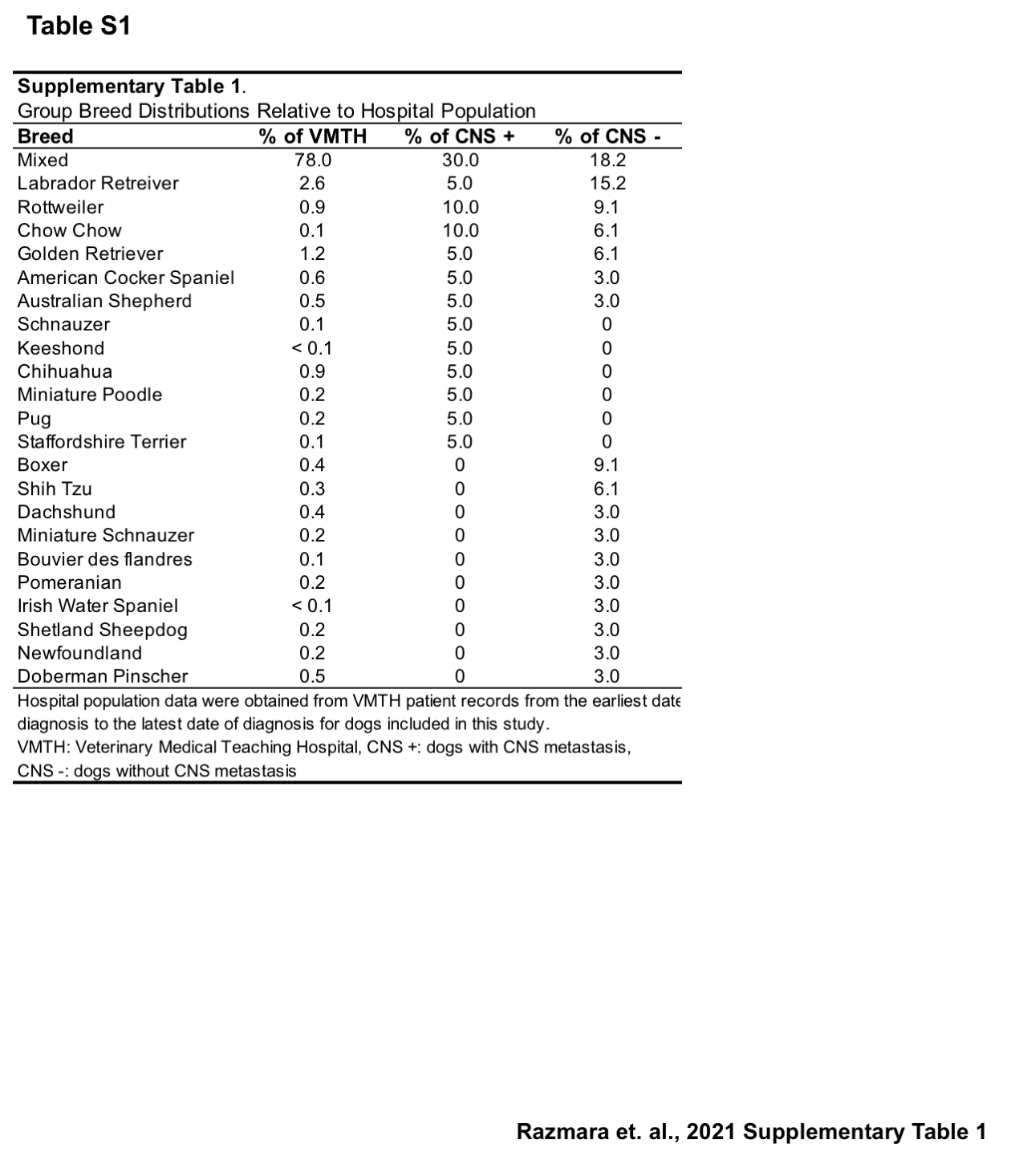

Supplement: Supplementary file 1 [file Image_1.tiff]

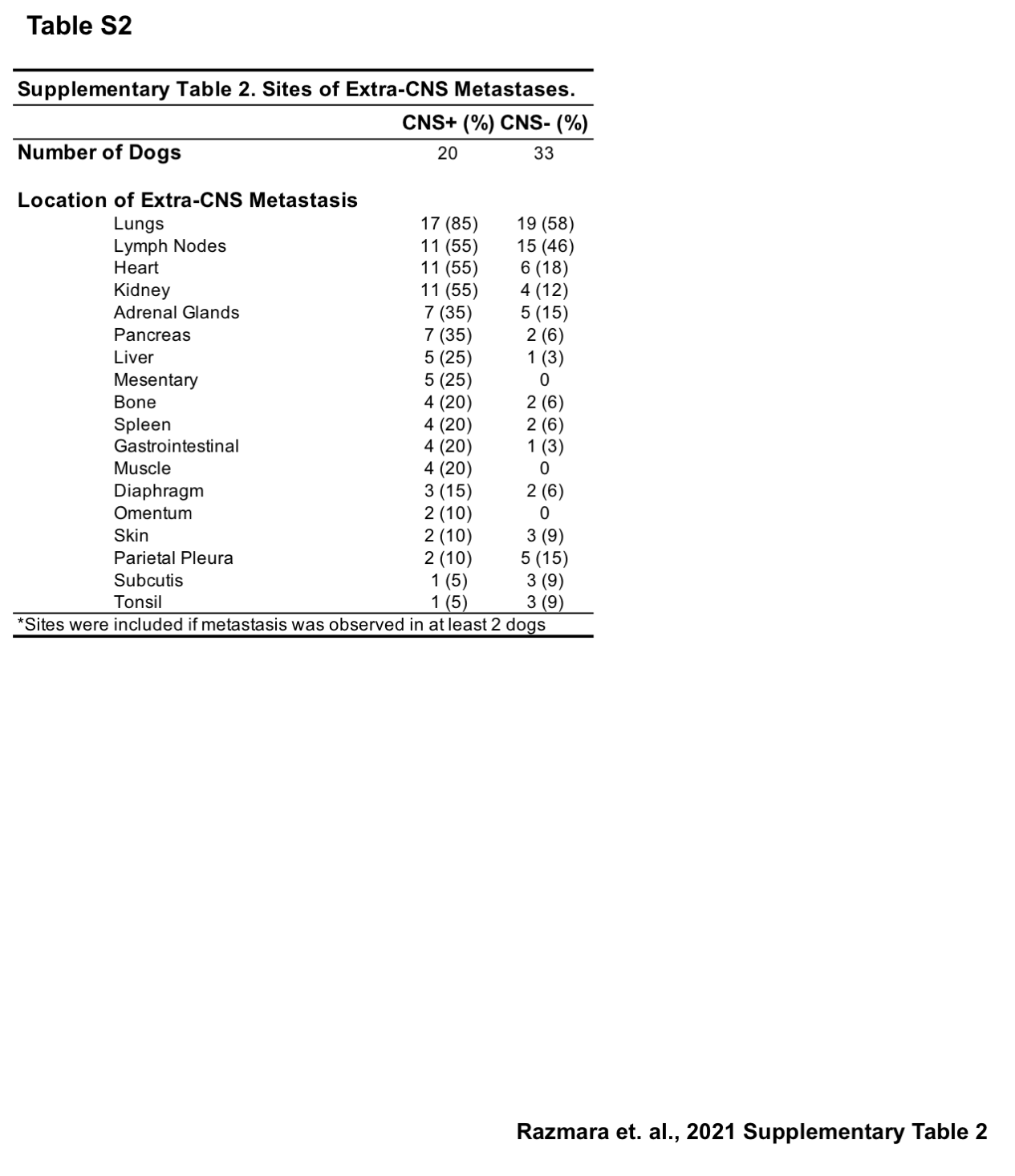

Supplement: Supplementary file 2 [file Image_2.tiff]

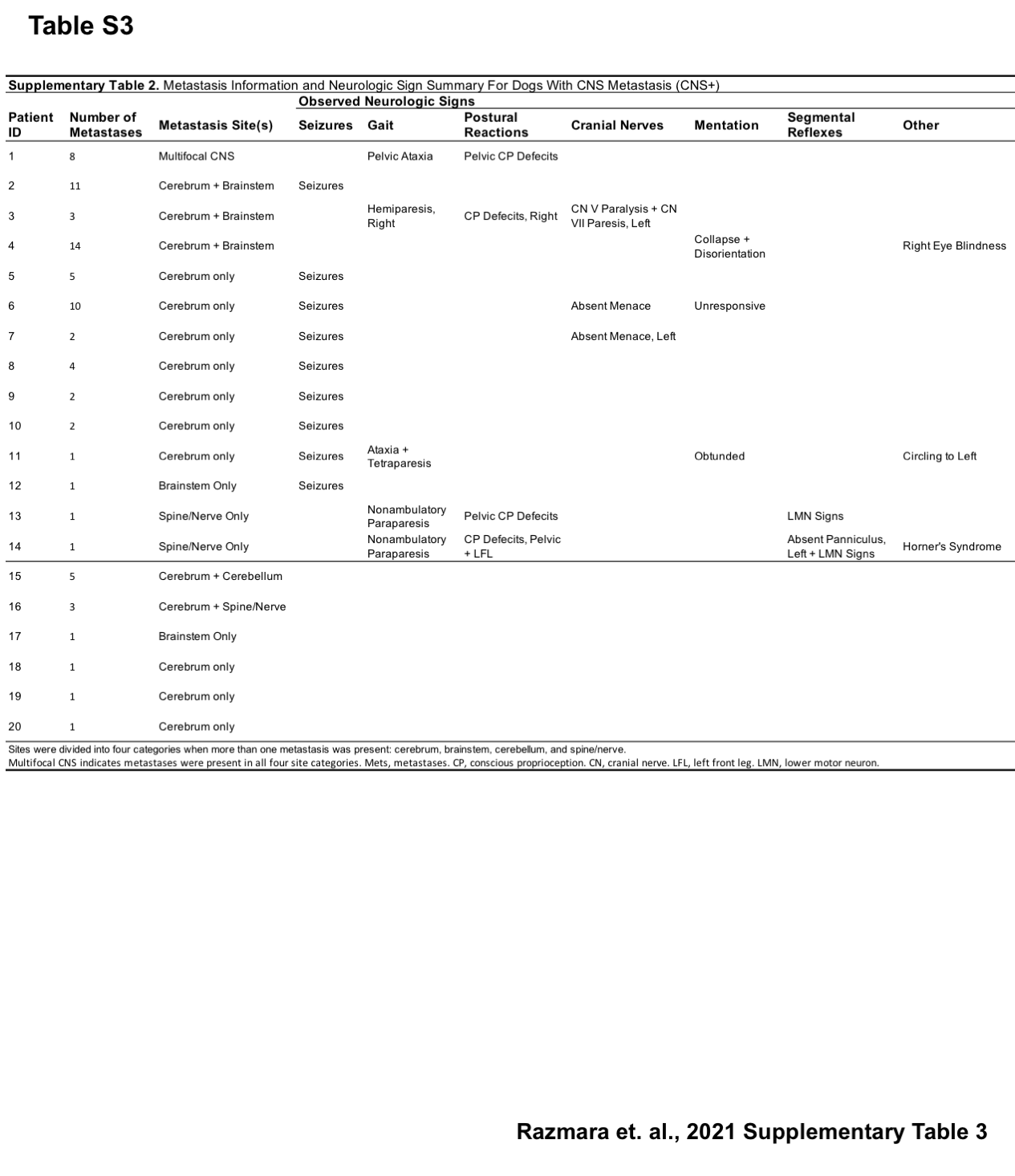

Supplement: Supplementary file 3 [file Image_3.tiff]
